# Acute and chronic antihypertensive effects of *Cinnamomum zeylanicum* stem bark methanol extract in L-NAME-induced hypertensive rats

**DOI:** 10.1186/1472-6882-13-27

**Published:** 2013-01-31

**Authors:** Paulin Nyadjeu, Elvine Pami Nguelefack-Mbuyo, Albert Donatien Atsamo, Telesphore Benoît Nguelefack, Alain Bertrand Dongmo, Albert Kamanyi

**Affiliations:** 1 Institute of Fisheries and Aquatic Sciences, University of Douala, P.O. Box 7236, Douala, Cameroon; 2 Department of Animal Biology, Laboratory of Animal Physiology and Phytopharmacology, University of Dschang, P.O. Box 67, Dschang, Cameroon; 3 Department of Animal Biology and Physiology, Laboratory of Animal Physiology, University of Yaounde I, P.O. Box 812, Yaounde, Cameroon; 4 Department of Animal Biology and Physiology, University of Douala, P.O. Box 24157, Douala, Cameroon

**Keywords:** Antihypertensive, Antihyperlipidemic, *Cinnamomum zeylanicum*, L-NAME-hypertensive rat, NO

## Abstract

**Background:**

Previous study showed that the aqueous extract of the stem bark of *Cinnamomum zeylanicum* possesses antihypertensive and vasodilatory properties. The present work investigates the acute and chronic antihypertensive effects of the methanol extract of *Cinnamomum zeylanicum* stem bark (MECZ) in L-NAME-induced hypertensive rats.

**Methods:**

The acute antihypertensive effects of MECZ (5, 10 and 20 mg/kg) administered intravenously were evaluated in rats in which acute arterial hypertension has been induced by intravenous administration of L-NAME (20 mg/kg). For chronic antihypertensive effects, animals were treated with L-NAME (40 mg/kg/day) plus the vehicle or L-NAME (40 mg/kg/day) in combination with captopril (20 mg/kg/day) or MECZ (300 mg/kg/day) and compared with control group receiving only distilled water. All drugs were administered *per os* and at the end of the experiment that lasted for four consecutive weeks, blood pressure was measured by invasive method and blood samples were collected for the determination of the lipid profile. The heart and aorta were collected, weighed and used for both histological analysis and determination of NO tissue content.

**Results:**

Acute intravenous administration of *C. zeylanicum* extract (5, 10 and 20 mg/kg) to L-NAME-induced hypertensive rats provoked a long-lasting decrease in blood pressure. Mean arterial blood pressure decreased by 12.5%, 26.6% and 30.6% at the doses of 5, 10 and 20 mg/kg, respectively. In chronic administration, MECZ and captopril significantly prevented the increase in blood pressure and organs’ weights, as well as tissue histological damages and were able to reverse the depletion in NO tissue’s concentration. The MECZ also significantly lower the plasma level of triglycerides (38.1%), total cholesterol (32.1%) and LDL-cholesterol (75.3%) while increasing that of HDL-cholesterol (58.4%) with a significant low atherogenic index (1.4 versus 5.3 for L-NAME group).

**Conclusion:**

MECZ possesses antihypertensive and organ protective effects that may result from its ability to increase the production of the endogenous NO and/or to regulate dyslipidemia.

## Background

Essential (or primary) hypertension accounts for approximately 90% to 95% of patients diagnosed with hypertension [1] and it is mainly caused by endothelial dysfunction which results from NO deficiency. In fact, it has been found that vascular endothelium of hypertensive patients produces less nitric oxide, a key regulator of cardiovascular system and metabolic homeostasis [2]. It acts as an antiatherogenic agent by inhibiting LDL oxidation, platelets aggregation, vascular smooth muscle proliferation, adhesion molecules expression and adhesion of monocytes to the endothelium [3,4]. Moreover, NO is known to be an anti-hypertrophic agent [5]. The inhibition of NO production by L-arginine analogue like N^ω^ Nitro-L- arginine methyl-ester (L-NAME) therefore results in arterial hypertension, dyslipidemia and histological damages. L-NAME-induced hypertension is thus a suitable model to study the cardiovascular effects of new active substances.

Despite the large number of antihypertensive drugs and the progresses made in the efficacy and tolerability of these agents, it is acknowledged that less than 25% of treated individuals achieve target blood pressure (BP) (i.e. <140/90 mmHg) [6]. Trial outcomes have shown that each agent is associated with relative benefits and drawbacks, often within the context of various patient characteristics such as age, co-morbidities and risk status [7]. The current trend towards lower BP goals suggests that more effective and better tolerated antihypertensive therapies will be needed, of which natural products can be considered as one of the potential sources. Indeed, various plant preparations have been used and claimed to have antihypertensive effects. The antihypertensive effects of some of these plants have been validated and others disproved. One of the plants used for the treatment of hypertension is *Cinnamomum zeylanicum* (Lauraceae). It is a native of Sri Lanka and south India [8]. Moderately sized tree, up to 2-16 m tall and 60 cm in diameter at breast height, *Cinnamomum zeylanicum* is covered with a thick, scabrous bark. The leaves are petiolate, opposite for the most part, coriaceous, obtusely pointed with three nerves; the lateral nerves vanishing as they approach the apex. When mature, the leaves are of a shiny green colour on their upper surface, and lighter colour beneath. The flowers are small, white, and arranged in axillaries and terminal panicles. The fruit is an oval berry, which adheres like the acorn to the receptacle, it’s larger than the black currant and when ripe has a bluish-brown surface, diversified with numerous white spots [9]. Traditionally, the stem bark of this plant is utilized in the management of various ailments in many Cameroonian communities. For example, it is used for the treatment of gastrointestinal disorders, typhoid fever, rheumatism and muscular pain. It is also used as an aphrodisiac and in cases of cardiovascular disorders including high blood pressure. Many pharmacological investigations carried out on *C. zeylanicum* demonstrated a number of useful effects including anti-nociceptive and anti-inflammatory [10], antidiabetic [11]. Phytochemical studies of *C. zeylanicum* revealed the presence of phenolic constituents in the fruits [12]. The analyses of the stem bark revealed the presence of tannin, mucilage, coumarine and essential oil. Cinnamaldehyde and camphor are major compounds present in stem-bark oil of this plant [13]. We previously showed that the aqueous extract of this plant possesses acute antihypertensive and vasorelaxant effects [14].

The present study attempted to evaluate the acute and chronic antihypertensive effects of the methanol extract of the stem bark of *C. zeylanicum* in L-NAME-induced hypertensive rats.

## Methods

### Plant collection and extraction

Fresh barks of *C. zeylanicum* were harvested in Njombe, Moungo Division in the Littoral Region of Cameroon in January 2007. The plant material was identified at the National Herbarium in Yaounde, in comparison to the voucher specimen number SRFC/22309. The barks were air-dried and ground to a fine powder. 800 g of the powder obtained were soaked at room temperature in 3 l of methanol for 48 h, with occasional shaking. After filtration, the filtrate was concentrated by evaporation at 70°C under reduced pressure on a rotary evaporator to afford 45 g of the methanol extract, corresponding to an extraction yield of 5.6%. This extract was dissolved in DMSO (4%) for daily used.

The major phytochemical groups were determined using the Lieberman Buchard, ferric chloride, copo of magnesium and Vanillin-sulphuric acid tests. Sterols, polyphenolic compounds, flavonoids, alkaloids and saponins were identified in the extract.

### Animals and experimental design

Male Wistar rats, aged 12–16 weeks and weighing 150 to 250 g were randomly selected from our colony. They were raised in the animal house of the Faculty of Sciences, University of Dschang, Cameroon. Animals were exposed to daily 12 h light–dark cycle with free access to a standard animal diet and tap water. The effects of *Cinnamomum zeylanicum* methanol extract (MECZ) were examined acutely and chronically *in vivo* on mean arterial blood pressure (MABP) of rats previously treated with L-NAME.

### Ethical consideration

Experimental protocols used in this study were approved by the Laboratory committee (Laboratory of Animal Physiology and Phytopharmacology, Department of Animal Biology, University of Dschang – Cameroon) according to the standard ethical guidelines for laboratory animal use and care as described in the European Community guidelines; EEC Directive 86/609/EEC, of the 24th November 1986 [15].

### Blood pressure and heart rate measurement

Blood pressure and cardiac frequency were determined by the invasive method. Brietly, Animals were anaesthetized by intraperitoneal administration of sodium thiopental at the dose of 50 mg/kg and a catheter was implanted in the femoral vein for drug administration. Another catheter was inserted in the left carotid artery for direct blood pressure measurement. Both catheters were filled with glucose-saline heparinized solution. The catheter inserted in the carotid artery was connected to a blood pressure transducer model Ugo Basile PRC 21k-10 coupled to an Ugo Basile Unirecord model 7050 for blood pressure recording. A stabilisation period of 30 minutes was observed before any recording. Heart frequency was determined by the use of pulse intervals.

### Experimental procedure

For acute antihypertensive study, a solution of L-NAME, an inhibitor of nitric oxide synthase was intravenously injection to normotensive Wistar rats (20 mg/kg) after the stabilization period at corresponding volume of 100 μl/100 g bw. MECZ was administered intravenously at the doses of 5, 10 or 20 mg/kg, twenty minutes after L-NAME administration, when the rise in blood pressure induced by L-NAME had reached the maximum [16].

In the chronic study, male Wistar rats were randomly divided into four groups of eight rats each. The first group (control) received a solution of DMSO 4% daily, while the second one (L-NAME group) received L-NAME (40 mg/kg/day) plus the vehicle. The third group was treated every day with a combined solution of L-NAME (40 mg/kg/day) and captopril (LN-Capto; 20 mg/kg/day) while the forth group, received a combination of L-NAME (40 mg/kg/day) and MECZ (LN-MECZ; 300 mg/kg/day). All the treatments were administered daily by gastric intubation on non-anesthetized animals for 4 weeks at the corresponding volume of 1 ml/100 g bw. The intubation was done daily, on non-anesthetized animals. The dose of the extract was determined based on previous results. At the end of the treatment, animals were anaesthetized by intraperitoneal administration of sodium thiopental (50 mg/kg) for blood pressure measurement. Immediately after blood pressure measurement, blood samples were collected from the abdominal artery, and centrifuged at 3000 rpm for 15 minutes. The plasma obtained was kept at −20°C for lipid assay. Thereafter, the heart and the thoracic aorta were collected, washed in saline and weighed. The heart was dissected out for the evaluation of the left ventricular mass. Three organs’ samples were fixed with 10% formalin in saline while the five others were used for NO evaluation.

### Biochemical analysis

The concentrations of total cholesterol (TC), high density lipoprotein (HDL) and triglycerides (TG) in plasma were determined spectrophotometrically using a commercially available kit Dialab and Helios Epsilon spectrophotometer. Low density lipoprotein (LDL) content was calculated from the other lipid parameters according to the [17] equation: LDL = TC–(TG/5)–HDL. The atherogenic index was calculated using the formula [18]: Atherogenic Index.

The left ventricle and aorta were homogenized in a carbonate buffer solution, and centrifuged at 4000 rpm for 15 minutes. The supernatant obtained was used to measure spectrophotometrically tissue concentration of nitric oxide (NO) as previously described by [19]. Briefly, 300 μl of sample were allowed to react with Griess reagent (1% sulfanilamide, 0.1% N-1-naphthylethylenediamine dihydrochloride and 2.5% phosphoric acid) at room temperature for 10 min, and then the absorbance was read at 530 nm. The NO concentration was determined by using NaNO_2_ standard curve.

### Aortic and left ventricular histological analysis

Fixed left ventricle and aorta were dehydrated and embedded in paraffin. 5 μm thick sections were mounted on glass slides. After deparaffinization and rehydration, they were stained with either Hematoxylin-Eosin or Van Gieson trichrome solutions in order to assess histological injuries and collagen accumulation in tissues. Histological analysis was performed with light microscope. The media thickness of aorta defined as the distance between the internal and external elastic lamina was determined using the following argument. Each photo have a bar corresponding to a given distance (aμm). Knowing that the magnification used was 400, the length (L) of each bar was then calculated using the formula: L = aμm/400.

### Drugs used

L-NAME was obtained from Fluka (Germany), sodium thiopental from Rotex Media (Germany), Heparin from Sanofi (France) and captopril was obtained from the Sahib Singh Agencies (India). All test solutions, including Krebs’ solution were freshly prepared in distilled water.

### Statistical analysis

Statistical analysis was performed using GraphPad Prism version 5.0. All results are expressed as means ± standard error of the mean. Data were analysed using one-way analysis of variance (*ANOVA*) followed by Tukeys’post test. Differences between means were considered to be significant when p < 0.05.

## Results

### Acute antihypertensive effects of the methanol extract of *Cinnamomum zeylanicum*

**Figure 1:**
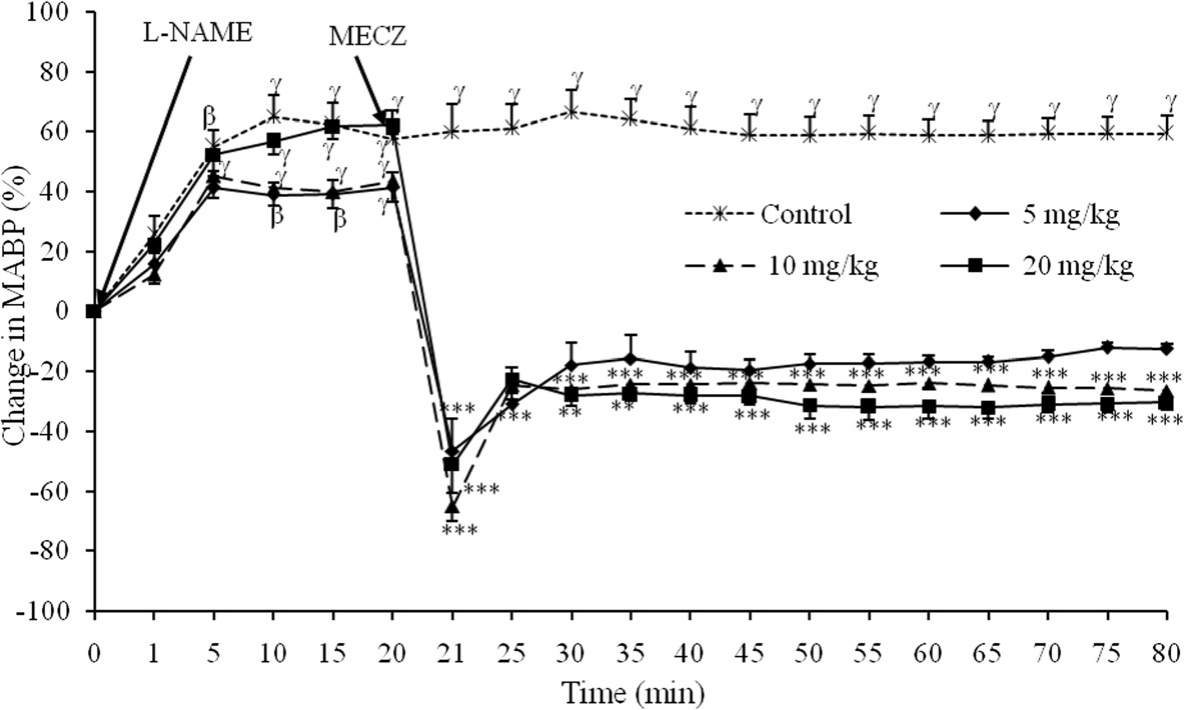
**Effect of the methanol extract from *Cinnamomum. zeylanicum *stem bark on the mean arterial blood pressure in L-NAME-induced hypertensive rats. **Each point represents the mean±SEM (n=5); ^α^p<0.05, ^β^p<0.01 and ^γ^p<0.001 significantly different compared to the initial value (0 minute). **p<0.01 and ***p<0.001 significantly different compared to the value 20 minutes after L-NAME administration.

Figure 1 shows the acute effects of MECZ on mean arterial blood pressure (MABP) examined *in vivo* in L-NAME induced hypertensive rats. L-NAME injected intravenously at the dose of 20 mg/kg in normotensive Wistar rats induced a sustained arterial hypertension which persisted for more than one hour and half with a MABP of 160.33 ± 3.82 mm Hg. Immediately following intravenous administration, MECZ induced a significant (p < 0.001) decrease in MABP. The lowest dose (5 mg/kg) reduced the elevated blood pressure by 46.4 ± 10.6%. The MABP dropped from 160 ± 4.02 mm Hg to 62.13 ± 11.28 mm Hg. In animals receiving the doses of 10 and 20 mg/kg, the MABP dropped suddenly after plant administration from 159.46 ± 5.77 mm Hg to 55.46 ± 7.31 mm Hg and from 176.66 ± 6.86 mm Hg to 83.46 ± 16.03 mm Hg; corresponding to an immediate reduction in MABP of 68.9 ± 4.8% and 50.7 ±9.5% respectively. The immediate fall in blood pressure induced by previous doses (5, 10 and 20 mg/kg) of the extract was followed by a long-lasting and dose-related antihypertensive effects that lasted for more than one hour with a corresponding decreases of 12.51 ± 1.7%, 27.23 ± 3.1% and 30.9 ± 3.1%, respectively.

### Chronic antihypertensive effects of the methanol extract of *Cinnamomum zeylanicum*

**Figure 2:**
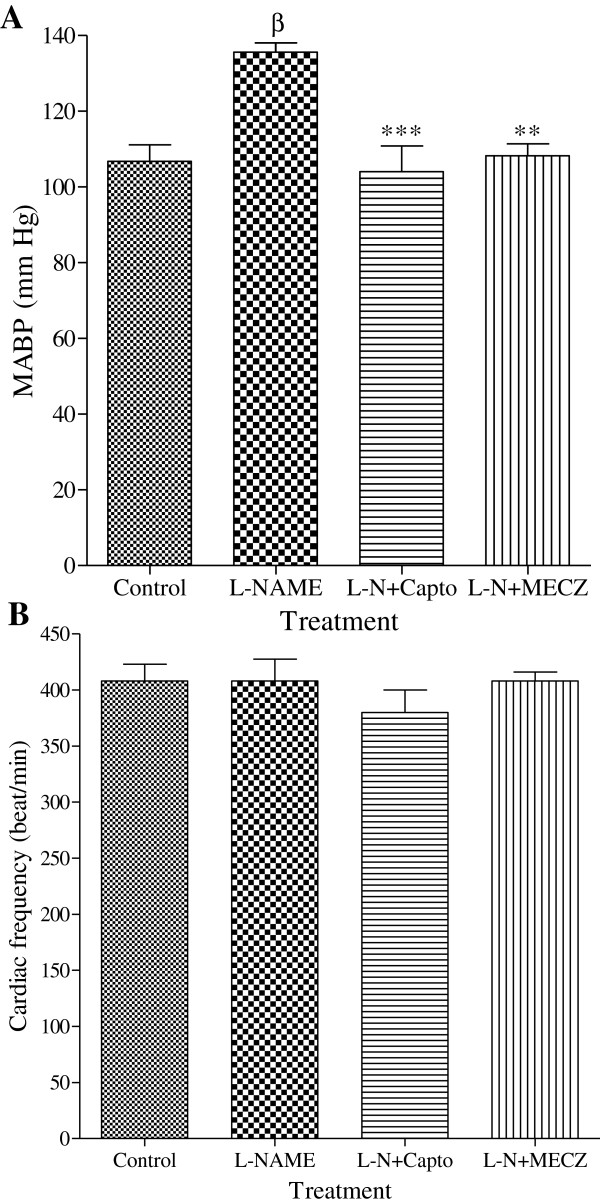
**Effects of the methanol extract of *Cinnamomum zeylanicum *on MABP and heart rate (B).** Values are Mean±SEM (n=5); ^β^p<0.01 compared to the control group; **p<0.01 and ***p<0.01 compared to the L-NAME group.

Animals that received only L-NAME (40 mg/kg/day) were hypertensive after four weeks of treatment. Concomitant administration of L-NAME with captopril (LN-Capto; 20 mg/kg/day) or MECZ (LN-MECZ; 300 mg/kg/day) prevented the installation of hypertension as depicted in Figure 2. MABP value was 135.6 mm Hg in L-NAME treated rats versus 104 ± 6.83 mm Hg and 108.26 ± 3.1 mm Hg in the LN-Capto and LN-MECZ groups respectively. However, no significant change in heart rate was observed between experimental groups (Figure 2).

### Long-term effects of the methanol extract of *Cinnamomum zeylanicum* on the final body weight and some organs weight

**Figure 3:**
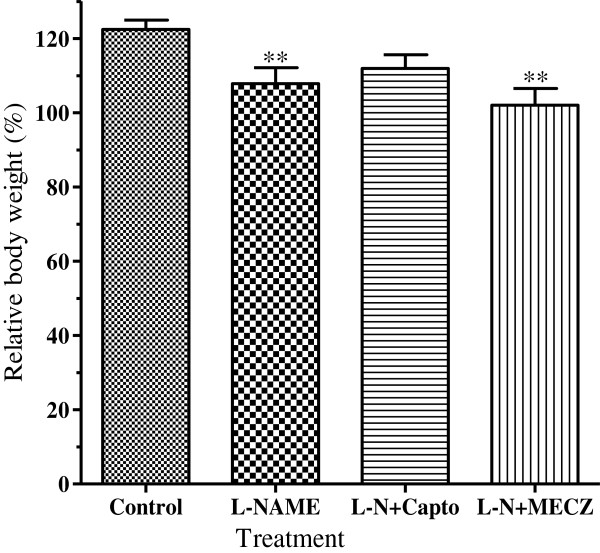
**Effects of chronic treatment on the rat relative body weight.** n=5; **p<0.01 significantly different compared to the control.

In the control group, the relative body weight increased by 122.5 ± 2.5% compared to initial value. This parameter was significantly low (p < 0.01) in L-NAME treated rats where the body weight increased only by 107.9 ± 4.3%. Contrary to captopril (112 ± 3.1%) slightly prevented the body weight loss induced by L-NAME as compared to the control group while the MECZ did not prevent body weight loss, with the relative body weight (102.1 ± 4.5%) significantly low (p < 0.01) compared to that of the control group (Figure 3).

**Figure 4:**
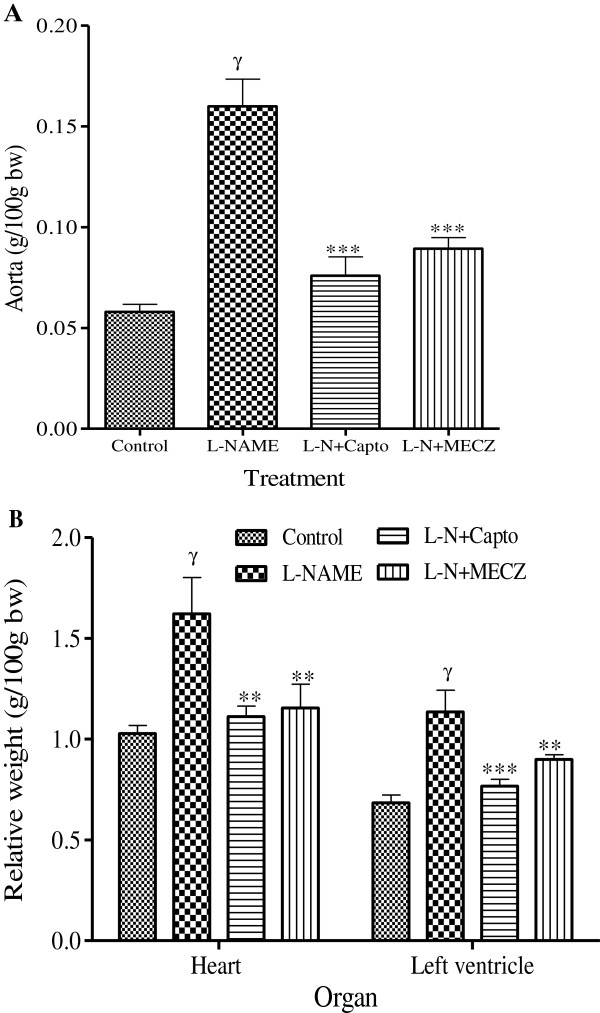
**Effects of the chronic treatment on the aorta, heart and left ventricle relative weight.** Values are Mean±SEM (n=5); ^γ^p<0.001 significantly different compared to the control group; **p<0.01 and ***p<0.001 significantly different compared to the L-NAME group.

In contrast to the effects observed on the body weight, long-term administration of L-NAME significantly (p < 0.001) increased both aorta (160.2 ± 15.3 mg/100 g bw versus 58 ± 4 mg/100g bw in the control group) and left ventricle (1.14 ± 0.11 g/100 g bw versus 0.68 ± 0.04 g/100 g bw in the control group) weights. The heart and vascular hypertrophy induced by L-NAME administration was significantly (p < 0.01) prevented by captopril and MECZ (Figure 4).

### Aortic and left ventricle histomorphologies

**Figure 5:**
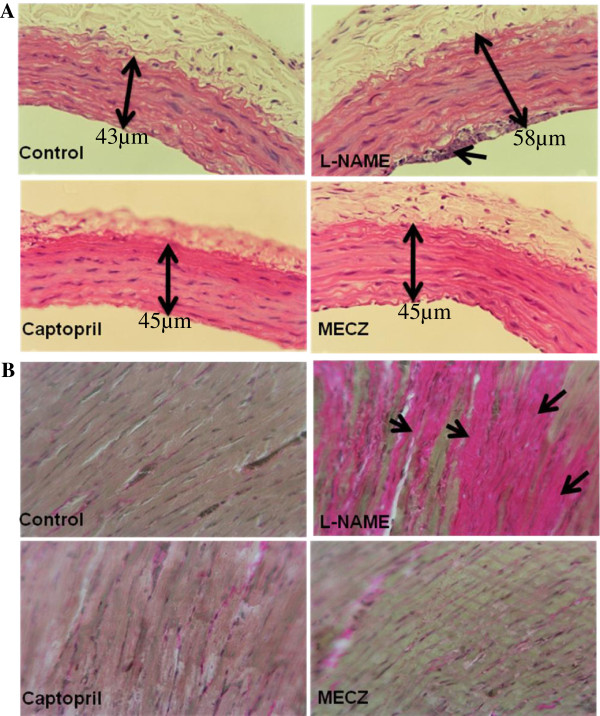
**Representative photographs showing aortic and left ventricular histomorphologies respectively stained with Hematoxylin & Eosin and Van Gieson trichrome.** Magnification: 400X.

Hematoxylin & Eosin staining revealed in L-NAME treated rat, an aortic media hypertrophy, characterized by an increase in the vessel section area (58 ± 0.11 μm versus 43 ± 0.03 μm in the control group). Moreover, cell accumulation on the endothelial wall was also noticed; which could lead to endothelial impairment. These vascular morphological damages were almost completely prevented by captopril (45 ± 0.21 μm versus 58 ± 0.11 μm in the L-NAME group) as well as the plant extract (45 ± 0.4 μm versus 58 ± 0.11 μm in the L-NAME group) (Figure 5A). Van Gieson trichrome revealed left ventricular structural changes characterized by fibrosis in L-NAME rats. In rats receiving either captopril or *C. zeylanicum* extract, the left ventricular histological injury was significantly attenuated (Figure 5B).

### Effects of the methanol extract of *C. zeylanicum* on tissue nitric oxide

**Figure 6:**
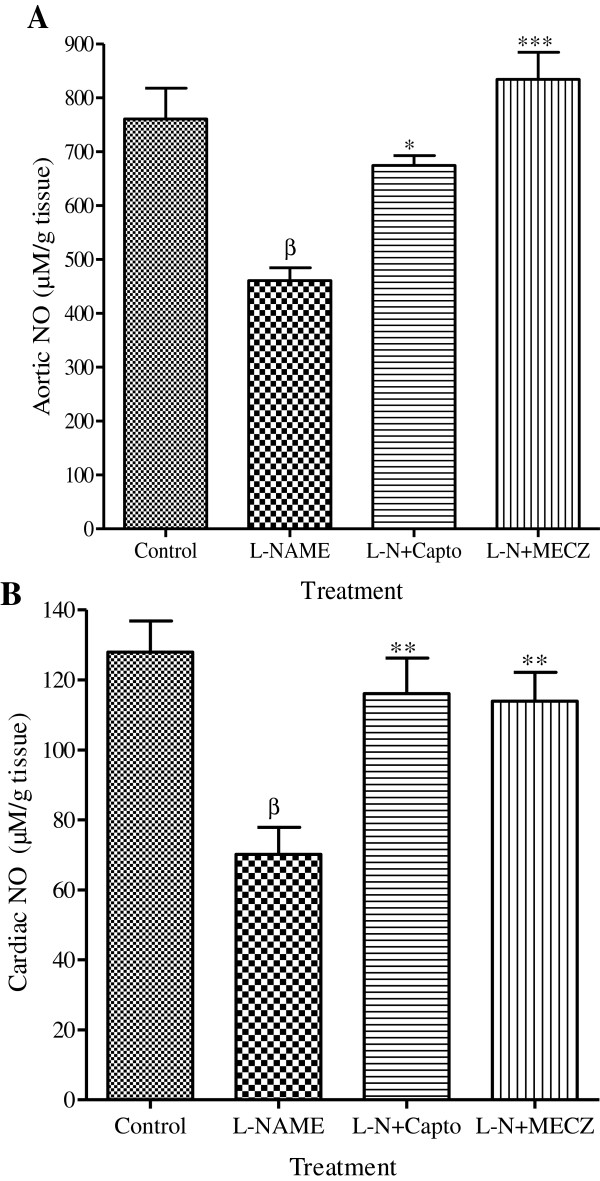
**Effects of the chronic treatment on aorta and cardiac NO content.** Values are Mean±SEM (n=5); ^β^p<0.01 significantly different compared to the control group; *p<0.05, **p<0.01 and ***p<0.001 significantly different compared to the L-NAME group.

Aortic and cardiac content in NO are represented in Figure 6. In rats treated only with L-NAME, both aortic and cardiac NO concentration decrease significantly (p < 0.01) by 39.4% and 45.2% respectively as compared to the control. The methanol extract of *C. zeylanicum* significantly prevented the deleterious effects of L-NAME in the tissue NO content. It increased by 47.7% and 38.4% the concentration of NO in the aortic and cardiac tissues respectively compared to the L-NAME group.

### Effects of the methanol extract from *C. zeylanicum* on the plasma lipid profile

The oral administration of L-NAME (40 mg/kg) to rats resulted in an elevated plasma lipid profile (Table 1). L-NAME treated rats showed significantly (p < 0.01) higher levels of plasma triglycerides, total cholesterol, LDL-cholesterol and significantly (p < 0.001) lower levels of HDL-cholesterol compared to the normal control rats. The methanol extract of the stem bark of *Cinnamomum zeylanicum* (300 mg/kg/day) significantly modulated the lipid profile in animals receiving concomitantly L-NAME, reducing triglycerides and total cholesterol by 38.10% and 32.1%, respectively. The extract increased the level of plasma HDL-cholesterol by 58.4% while reducing that of LDL-cholesterol by 75.3%, with and atherogenic index closer to that of the control untreated rats.

**Table 1:** **Effects of the methanol extract from *C. zeylanicum *on lipid profile in L-NAME hypertensive rats**

**Treatment**	**Triglycerides (mg/dl)**	**Total cholesterol (mg/dl)**	**HDL-cholesterol (mg/dl)**	**LDL-cholesterol (mg/dl)**	**Atherogenic index**
Control	53.48 ± 3.36	50.63 ± 2.07	48.76 ± 3.13	5.51 ± 3.68	1.06 ± 0.31
L-NAME	81.10 ± 3.80^β^	83.13 ± 4.02^γ^	17.57 ± 2.82^β^	38.52 ± 4.29^β^	5.35 ± 0.48^γ^
L-N + Capto	45.74 ± 3.36**	48.12 ± 2.09***	40.40 ± 3.32**	4.50 ± 3.96***	1.50 ± 0.70***
L-N + MECZ	50.16 ± 3.33*	56.46 ± 5.29***	42.26 ± 3.83**	9.50 ± 3.08***	1.41 ± 0.18***

n = 5; ^β^p < 0.01 and ^γ^p < 0.001 significantly different compared to the control; ^*^p < 0.05, ^**^p < 0.01 and ^***^p < 0.001 significantly different compared to L-NAME. L-NAME group received only L-NAME 40 mg/kg/day. L-N + Capto received L-NAME plus captopril (20 mg/kg/day) while L-N + MECZ received L-NAME plus extract (300 mg/kg/day).

## Discussion

This study investigated the acute and chronic antihypertensive effects of the stem bark methanol extract of *Cinnamomum zeylanicum* in L-NAME-induced hypertensive rats and its effects on lipid metabolism in chronic hypertensive rats. In this model, inhibition of NO-synthase permits the evaluation of the nitric oxide pathway, a physiologically important vasodilator, playing a major role in the regulation of systemic hemodynamic [20]. Acute intravenous administration of *Cinnamomum zeylanicum* methanol extract to L-NAME-induced hypertensive rats caused a significant decrease in mean arterial blood pressure (MABP) that last more than an hour after administration. In animals treated chronically with L-NAME, arterial hypertension associated with both cardiovascular hypertrophy and histological damages were observed. It is well known that L-NAME, administered either acutely by intravenous route or chronically by the oral induced sustained hypertension [16,21,22]. But Chaswal *et al.*[23] showed that the acute hypertension provoked by L-NAME administration may not involve the renin angiotensin system which is at least partially responsible for the chronic response. The fact that MECZ could reduce both the acute and chronic hypertension induced by L-NAME indicates that it may use another pathway. Previous studies using the aqueous extract of *C. zeylanicum* have demonstrated that this plant possesses both endothelium-dependent and -independent vasodilating properties [14]. Therefore, we postulate that the acute antihypertensive effect of our plant extract might be mainly due to its ability to reduce the peripheral resistance via its vasodilating activities even though its effect on the renin-angiotensin system, which plays a pivotal role in the development of chronic L-NAME hypertension, cannot be rule out. It is also well known that in this model of experimental arterial hypertension, the sympathetic system tone and baroreflex to phenylephrine are significantly increased [23]. MECZ could also interfere with this system. Permanent increase in systemic blood pressure often lead to cardiovascular hypertrophy [24,25]. In fact, in the present study, it was found that chronic treatment with L-NAME increases the cardiac and aorta weights. Additionally, histological analysis showed the thickness of the vascular wall as well as fibroblast infiltration in the myocardium. These effects were significantly reversed by MECZ and captopril. Results with captopril corroborate many other previous works [26,27] and further confirm the involvement of the renin-angiotensin system in the development of this model of arterial hypertension. As there is a tight relationship between the renin-angiotensin system and the sympathetic system, the inhibition of one may lead to the inhibition of the other, as it can be clearly see in the case of captopril. So, no obvious mechanism can be attributed to MECZ. Another interesting fact is that MECZ was able to increase NO production in cardiovascular organs, namely the aorta and the heart. Thus, the antihypertensive as well as the antihypertrophic effects of MECZ may be at least partially attributed to the increase in NO production, which is known as an inhibitor to cell proliferation. Moreover, by stimulating cardiovascular NO synthesis, the plant extract could induces vasorelaxation, improves arterial wall compliance and control of blood pressure. Hypertension and hyperlipidemia are two concomitant cardiovascular risk factors. Therefore, in order to reduce cardiovascular risk, it is important to regulate hypertension as well as dyslipidemia, which is known as a qualitative or quantitative modification of one or several parameters of the plasma lipids [28]. The results of this study indicated a dyslipidemia in rats receiving L-NAME. The dyslipidemia observed was characterized by hypertriglyceridemia, hypercholesterolemia, and high rate of LDL-cholesterol (LDL-C) coupled with low level of HDL-cholesterol (HDL-C. The dyslipidemia observed in L-NAME-induced hypertensive rats, was significantly managed by both the captopril and MECZ with the concentration of triglycerides, total cholesterol, LDL-C or HDL-C, closer to that of normal rats. These results clearly demonstrated both the hypotriglyceridemic and hypocholesterolemic properties of the plant extract, which might contribute to the cardiovascular proptective effects of MECZ. The pharmacological activities of MECZ might be explained by the presence of phytochemical constituents like flavanoids and saponins in the plant. These bioactive phytomolecules are known to possess vasorelaxant, antihypertensive and antihyperlipidemic effects [29-31].

## Conclusion

In conclusion, this study demonstrated the acute and chronic antihypertensive properties of the methanol extract of *Cinnamomum zeylancum* stem bark in NO-deficient hypertensive rats. The acute antihypertensive effects might be partially due to the reduction of the peripheral arterial resistance by thwarting the acute inhibitory effect of L-NAME on eNOS. While, the long-term antihypertensive effects could be attributed to the ability of the plant active phytomolecules to inhibit cardiovascular remodelling, to improve arterial wall compliance and to prevent endothelial impairment through its antidyslipidemic effects.

## Competing interests

The authors declare that they have no competing interests.

## Authors’ contributions

PN: Prepared the extract, carried out the assays and drafted the manuscript. EPNM and SLW: Literature search and corrected the manuscript for publication. ADA: Helped in the experimental work. TBN and ABD: Supplied the materials, coordinated the study and refined the manuscript for publication. AK: Supervised the work and refined the manuscript for publication. All authors read and approved the final manuscript for publication.

## Pre-publication history

The pre-publication history for this paper can be accessed here:

http://www.biomedcentral.com/1472-6882/13/27/prepub
